# Dietary β-Carotene on Postpartum Uterine Recovery in Mice: Crosstalk Between Gut Microbiota and Inflammation

**DOI:** 10.3389/fimmu.2021.744425

**Published:** 2021-11-24

**Authors:** Xizi Yang, Ziyu He, Ruizhi Hu, Jiahao Yan, Qianjin Zhang, Baizhen Li, Xupeng Yuan, Hongfu Zhang, Jianhua He, Shusong Wu

**Affiliations:** ^1^ Hunan Collaborative Innovation Center for Utilization of Botanical Functional Ingredients, College of Animal Science and Technology, Hunan Agricultural University, Changsha, China; ^2^ Department of Food Science and Biotechnology, Faculty of Agriculture, Kagoshima University, Kagoshima, Japan; ^3^ Pig Breeding Research Insititute, Hunan Xinguang’an Agricultural Husbandry Co., Ltd., Changsha, China; ^4^ State Key Laboratory of Animal Nutrition, Institute of Animal Sciences, Chinese Academy of Agricultural Sciences, Beijing, China

**Keywords:** β-carotene, postpartum uterine recovery, gut microbiota, inflammation, reproductive performance

## Abstract

As the precursor of vitamin A, β-carotene has a positive effect on reproductive performance. Our previous study has shown that β-carotene can increase antioxidant enzyme activity potentially through regulating gut microbiota in pregnant sows. This study aimed to clarify the effect of β-carotene on reproductive performance and postpartum uterine recovery from the aspect of inflammation and gut microbiota by using a mouse model. Twenty-seven 6 weeks old female Kunming mice were randomly assigned into 3 groups (n=9), and fed with a diet containing 0, 30 or 90 mg/kg β-carotene, respectively. The results showed that dietary supplementation of β-carotene reduced postpartum uterine hyperemia and uterine mass index (*P*<0.05), improved intestinal villus height and villus height to crypt depth ratio, decreased serum TNF-α and IL-4 concentration (*P*<0.05), while no differences were observed in litter size and litter weight among three treatments. Characterization of gut microbiota revealed that β-carotene up-regulated the relative abundance of genera *Akkermansia*, *Candidatus Stoquefichus* and *Faecalibaculum*, but down-regulated the relative abundance of *Alloprevotella* and *Helicobacter*. Correlation analysis revealed that *Akkermansia* was negatively correlated with the IL-4 concentration, while *Candidatus Stoquefichus* and *Faecalibaculum* had a negative linear correlation with both TNF-α and IL-4 concentration. On the other hand, *Alloprevotella* was positively correlated with the TNF-α, and *Helicobacter* had a positive correlation with both TNF-α and IL-4 concentration. These data demonstrated that dietary supplementation of β-carotene contributes to postpartum uterine recovery by decreasing postpartum uterine hemorrhage and inhibiting the production of inflammatory cytokines potentially through modulating gut microbiota.

**Graphical Abstract:**
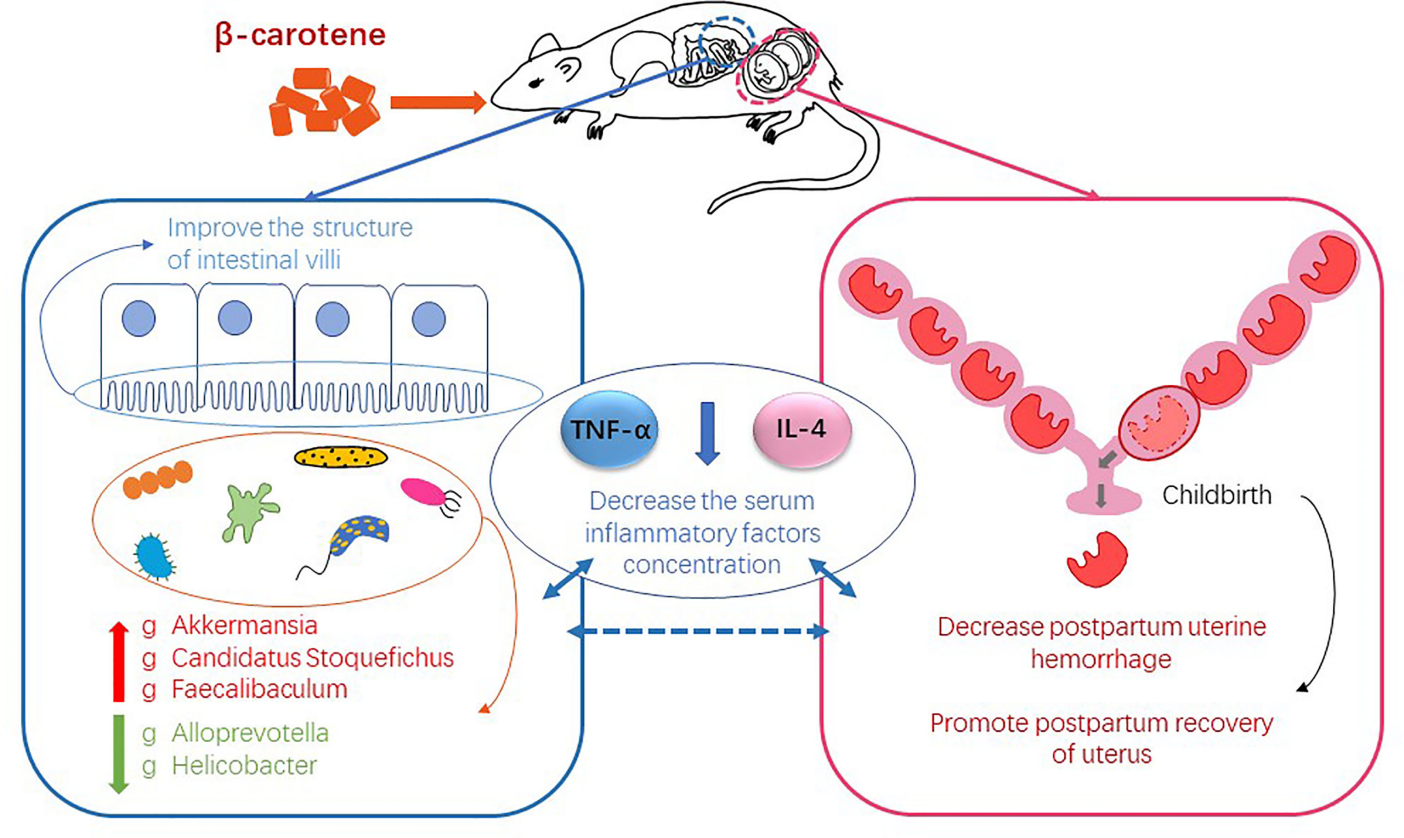


## Introduction

Regular consumption of fruits and vegetables are believed to decrease the incidence of chronic disease (1), and the beneficial health effects may attribute to secondary plant compounds such as carotenoids (2, 3). β-carotene is one of the main dietary carotenoids, and it can be converted to vitamin A in cells of intestinal mucosa, liver, uterus, and ovaries (4), although the mechanisms are not clear (5). β-carotene metabolism plays an important role during mammalian embryonic development (6). Recent studies have suggested that dietary β-carotene supplementation during late pregnancy has benefit to the birth weight of offspring in sows (7, 8), and injection of β-carotene can increase the litter size (9). Moreover, β-carotene has been reported to suppress inflammatory responses in macrophages by inhibiting NF-κB translocation (10), which can induce the expression of pro-inflammatory genes (11) and promote inflammatory diseases (12). The uterus is an important reproductive organ with fibromuscular characteristics (13). During childbirth, sustain stretching muscle tissue, resulting tissue damage and adverse outcomes (14), promote postpartum recovery of uterus, which is beneficial to the mother. A previous study has shown that β-carotene can reduce the incidence of retained placenta and metritis in cats (15), and uterine involution may have been more complete and that uterine inflammation may have been reduced in cows which received the β-carotene (16). The above results suggested that β-carotene may contribute to postpartum recovery.

The diverse collection of microorganisms that inhabit the gastrointestinal tract, collectively called the gut microbiota, profoundly influences many aspects of host physiology, including nutrient metabolism, resistance to infection and immune system development (17). Previous studies have suggested that pregnancy is associated with a significant alteration of the gut microbiota, which can affect the physiological state and metabolism of host (18). As a “metabolic organ,” gut microbiota undergo adaptive changes during pregnancy, especially in the third trimester (19). Gut microbiota can influence intestinal permeability, thereby promoting translocation of bacterial products to induce inflammation (20), which occurs throughout pregnancy (21). Our previous studies have also revealed that gut microbiota is closely correlated with inflammation and intestinal mucosal barrier function (22), and dietary supplementation of β-carotene can regulate host gut microbiota (8).

On the basis of previous studies, this study aimed to investigate the effect of β-carotene on the reproductive performance and postpartum uterine recovery, and challenged to elucidate the possible mechanism from the perspective of gut microbiota by using a mouse model.

## Materials and Methods

### Animals, Diets and Experimental Design

All animal protocols were performed in accordance with guidelines of the Animal Care and Use Committee of Hunan Agricultural University (Permission No. 2020041). Twenty-seven female and twenty-seven male Kunming mice aged 6 weeks were purchased from Hunan SJA Laboratory Animal Co, Ltd. (Changsha, Hunan, China). Each mouse was housed individually in cages with wood shavings bedding under controlled light (12-h light/day) and temperature (23°C), and free access to water and feed. After acclimatization for 1 week, female mice were randomly assigned to 3 groups (n=9), and fed with a basal diet (control group, CLT), a diet containing 30 mg/kg of β-carotene (β-carotene low dose group, CARL), or a diet containing 90 mg/kg of β-carotene (β-carotene high dose group, CARH), based on our previous study, respectively. Male mice were put in female cages from 8:00 PM to 8:00 AM until the copulatory plug was observed, and recorded the date.

### Parturition Record and Sample Collection

Food intake was recorded every three days during pregnancy. On the day of delivery, the mice were anesthetized and sacrificed, and the date, uterine mass, as well as the number of mice pups born live and the birth weight were recorded. Ileum and uterine samples were fixed in 4% para-formaldehyde solution immediately, liver, the rest of ileum and uterine organs and sample of cecum contents were collected, immediately put them in liquid nitrogen for quick freezing, and collected blood samples, after standing for 30 minutes, centrifuged at 1500 g for 10 minutes to obtain serum and store at -80°C.

### Histology

For hematoxylin and eosin (H&E): Ileum and uterine tissues were fixed in formalin overnight, dehydrated by titrating in ethanol (50%), and submitted to Wuhan Servicebio Technology Co., Ltd (Wuhan, Hubei, China) for paraffin embedding, sectioning, then put the slices into xylene I for 20 min, xylene II for 20 min, absolute ethanol I for 5 min, absolute ethanol II for 5min, 75% alcohol for 5 min, and wash with tap water. The slices were stained with hematoxylin dye solution for 3-5 min, washed with tap water, differentiated with differentiation solution, washed with tap water, hematoxylin-eosin stain returned to blue, and rinsed with running water. The slices were dehydrated with 85% and 95% gradient alcohol for 5 min, and stained with eosin for 5 min. Put absolute ethanol I for 5 min, absolute ethanol II for 5 min, absolute ethanol III for 5 min, dimethyl I for 5 min, xylene II for 5 min, transparent and sealed with neutral gum. The microscopes, image acquisition and length measurement were performed using MShot biological microscope ML31.

### Measurement of Antioxidant Indexes of Liver

Liver samples were accurately weighed, normal saline was added according to mass: volume = 1:9 and homogenized, then centrifuged at 4000 rpm for 10 min at 4°C. Total antioxidant capacity (T-AOC) and the level of MDA reflected by thiobarbituric acid reactive substances (TBARS) were measured using respective assay kits (Nanjing jiancheng Bioengineering Institute, Nanjing, China). Protein content was determined by using a BCA protein assay kit (Nanjing jiancheng Bioengineering Institute, Nanjing, China). All values were normalized by the protein content of the same sample.

### Measurement of Inflammatory Cytokines

Serum tumor necrosis factor-α (TNF-α) and interleukin-4 (IL-4) concentration were detected using ELISA kits (Nanjing jiancheng Bioengineering Institute, Nanjing, China) according to the manufacturer’s manual.

### Characterization of Gut Microbiota

Gut microbiota was characterized by 16S rRNA gene sequencing as described previously (8). Briefly, Total DNA was extracted from cecum contents by using a DNA Isolation Kit (MoBio Laboratories, Carlsbad, CA, USA) following the manufacturer’ s manual. The V3–4 hypervariable region of the bacterial 16S rRNA gene was amplified with the primers 338F (5′-ACTCCTACGGGAGGCAGCA-3′) and 806R (5′-GGACTACHVGGGTWTCTAAT-3′). The PCR was carried out on a Mastercycler Gradient (Eppendorf, Germany) using 25 µL reaction volumes, containing 12.5 µL KAPA 2G Robust Hot Start Ready Mix, 1 µL Forward Primer (5 µmol/L), 1 µL Reverse Primer (5 µmol/L), 5 µL DNA (total template quantity is 30 ng), and 5.5 µL H2O. Cycling parameters were 95°C for 5min, followed by 28 cycles of 95°C for 45 s, 55°C for 50 s, and 72°C for 45 s with a final extension at 72°C for 10min. The PCR products were purified using a QIAquick Gel Extraction Kit (QIAGEN, Germany), and quantified using Real Time PCR, and sequenced on Miseq platform at Allwegene Technology Inc., Beijing, China. Qualified reads were separated using the sample-specific barcode sequences and trimmed with Illumina Analysis Pipeline Version 2.6, and then the dataset was analyzed using QIIME (Version 1.8.0).

### Statistical Analysis

Results were expressed as means ± SD. The significant differences between groups were analyzed by one-way ANOVA tests, followed by Fisher’ s least significant difference (LSD) tests with the SPSS statistical software (IBM SPSS Statistics, version 19). Probabilities that were <0.05 were regarded as significant. Bivariate associations between serum TNF-α concentration and the abundance of microbiota, bivariate associations between serum IL-4 concentration and the abundance of microbiota were assessed by Spearman rank correlations, respectively. Then the linear relationship between the abundance of genus regulated significantly by β-carotene and inflammatory factors were analyzed using linear regression method. Principal component analysis (PCA) can reflect the difference and distance between samples by analyzing the composition of OUT (97% similarity) of different samples. Variance decomposition is used to reflect the difference of multiple groups of data on the two-dimensional coordinate map, and the coordinate axis takes two eigenvalues that can reflect the maximum variance value. If the sample composition is more similar, the closer the distance reflected in the PCA diagram.

## Results

### The Effect of β-Carotene on Litter Performance of Mice

As shown in **Table 1**, there was no significant difference in average daily food intake during pregnancy in mice, and no significant difference in litter size, weight and offspring average weight between CTL, CARL and CARH group.

**Table 1:** Effect of β-carotene on litter performance of mice.

Item	CTL	CARL	CARH	P-value
Average daily food intake, g	4.68 ± 0.19	4.49 ± 0.23	4.52 ± 0.14	0.083
Litter size	13.56 ± 2.46	14.11 ± 3.18	15.22 ± 1.56	0.367
Litter weight, g	23.71 ± 3.69	24.43 ± 4.35	26.82 ± 2.48	0.179
Offspring average weight, g	1.76 ± 0.10	1.75 ± 0.15	1.77 ± 0.10	0.975

CTL, a basal diet; CARL, a basal diet containing 30 mg/kg β-carotene; CARH, a basal diet containing 90 mg/kg β-carotene. Values are means ± SD (n = 9).

### The Effect of β-Carotene on Uterine Morphology and Inflammation

**Figure 1:**
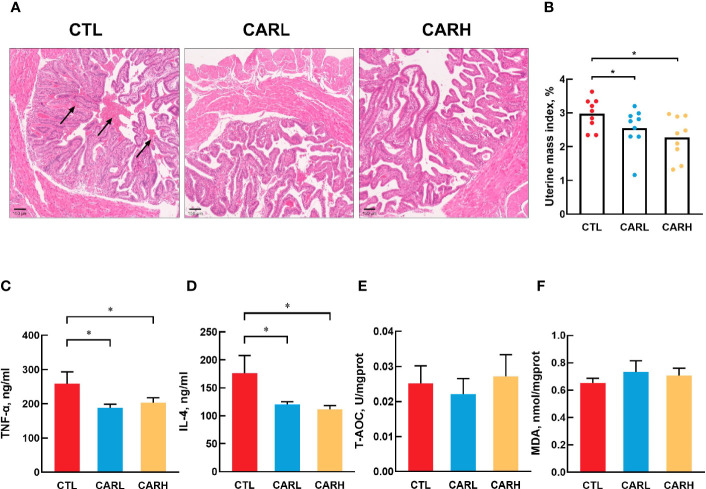
Effect of β-carotene on uterine morphology and serum biomarkers. Representative images of hematoxylin and eosin (H&E) stained uterine section **(A)**, uterine mass index=uterine mass/body weight **(B)**, serum TNF-α **(C)**, IL-4 **(D)** concentration, total antioxidant capacity of liver **(E)**, MDA content **(F)**. CTL, a basal diet; CARL, a basal diet containing 30 mg/kg β-carotene; CARH, a basal diet containing 90 mg/kg β-carotene. Data were shown as means ± SD (n = 9). *P < 0.05.

As shown in **Figure 1**, there is more intrauterine blood stasis in CTL group than that in the CARL and CARH group (A). Meanwhile, β-carotene significantly decreased the uterine mass index (*P*<0.05) (B), serum TNF-α (*P*<0.05) (C) and IL-4 concentration (*P*<0.05) (D), as compared with CTL group. However, there was no difference on T-AOC (E) and MDA level (F) in liver among the three groups.

### The Effect of β-Carotene on Mucosal Morphology

**Figure 2:**
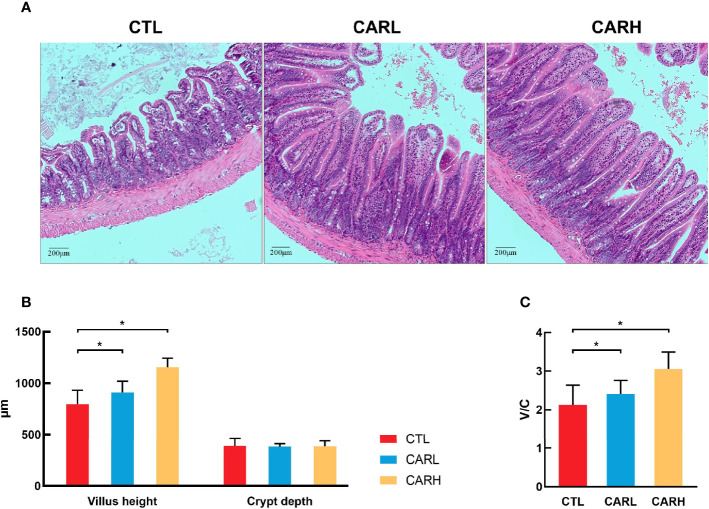
The effect of β-carotene on gut morphology. Representative images of ileum mucosa section stained by hematoxylin and eosin (H&E) **(A)**, villus height and crypt depth of ileum **(B)**, ratio of villus height to crypt depth **(C)** were analyzed. CTL, a basal diet; CARL, a basal diet containing 30 mg/kg β-carotene; CARH, a basal diet containing 90 mg/kg β-carotene. Data were shown as means ± SD (n = 9). *P < 0.05.

Mucosal crypt-villus axis affects the metabolic and inflammatory response (23), and the damage of intestinal cause to increase the intestinal permeability and inflammatory responses (24), thus the mucosal morphology of mice was observed. As shown in **Figure 2**, dietary supplementation of β-carotene improved the integrity of mucosa (A), and significantly increased villus height (*P*<0.05) (B) and ratio of villus height to crypt depth (*P*<0.05) (C).

### The Effect of β-Carotene on Gut Microbiota

**Figure 3:**
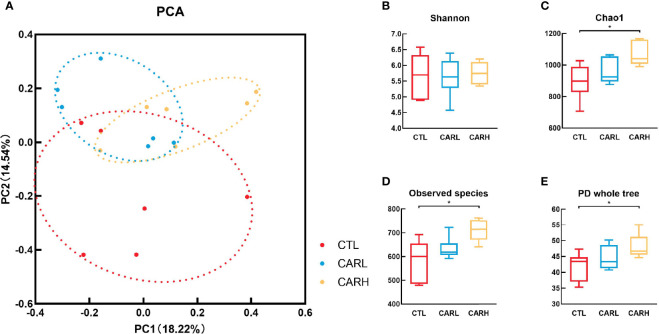
Effects of β -carotene on microbiota diversity. Effect of β-carotene on genus-based (n=6) PCA **(A)**, Shannon index **(B)**, Chao 1 index **(C)**, Observed species index **(D)** and PD whole tree index **(E)**. CTL, a basal diet; CARL, a basal diet containing 30 mg/kg β-carotene; CARH, a basal diet containing 90 mg/kg β-carotene. Data were shown as means ± SD (n = 6). Anosim: R = 0.135, *P*= 0.049, *P < 0.05.

The result of principal component analysis (PCA) of gut microbiota has been shown in **Figure 3A**, β-carotene had a significant effect on the structure of gut microbiota (Anosim: R=0.135, *P*=0.049). β-carotene had limited effect on the Shannon index (**Figure 3B**), however, the Chao 1 index (**Figure 3C**), Observed species index (**Figure 3D**) and PD whole tree index (**Figure 3E**) of CARH group were significantly higher than that of CTL group (*P*<0.05).

**Figure 4:**
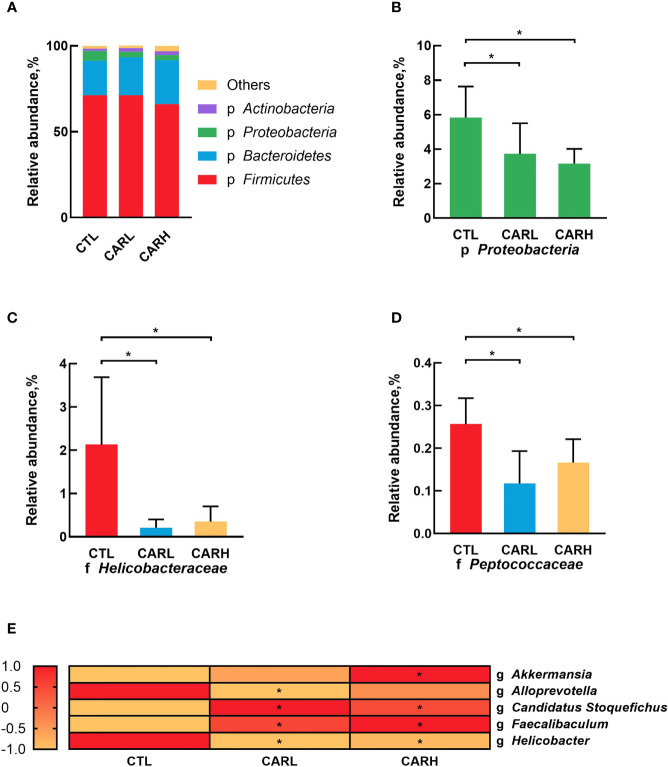
Modulation of gut microbiota by β-carotene. Effect of β-carotene on the relative abundance of main microbes at the phylum level **(A)**, *proteobacteria* phylum **(B)**, *Helicobacteraceae* family **(C)** and *Peptococcaceae* family **(D)**, and main microbial genera **(E)** were analyzed by 16S rRNA gene sequencing. CTL, a basal diet; CARL, a basal diet containing 30 mg/kg β-carotene; CARH, a basal diet containing 90 mg/kg β-carotene. Data were shown as means ± SD (n = 6). *P < 0.05.

The analysis of gut microbiota at the phyla level showed that β-carotene significantly down-regulated the relative abundance of *Proteobacteria* (*P*<0.05) (**Figures 4A, B**), while β-carotene significantly decreased the relative abundances of *Helico-bacteraceae* (**Figure 4C**) and *Peptococcaceae* (**Figure 4D**) at the family level (*P*<0.05), as compared with CTL group. At the genus level, β-carotene up-regulated the relative abundance of *Akkermansia*, *Candidatus Stoquefichus* and *Faecalibaculum*, and down-regulated the relative abundances of *Alloprevotella* and *Helicobacter* (*P*<0.05), which were selected from top 40 genera that affected by β-carotene (**Figure 4E**).

### Correlation Analysis of Gut Microbiota and Serum Inflammatory Cytokines

**Figure 5:**
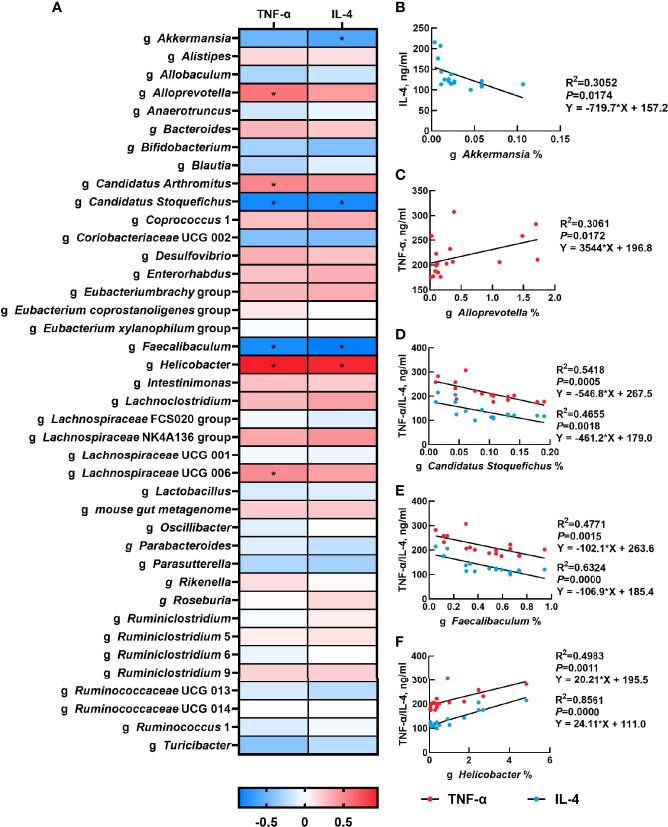
Correlation analysis of gut microbiota and serum inflammatory cytokines. The correlation analysis between the abundance of top 40 microbial genera and serum inflammatory cytokines concentration **(A)**, and the linear correlation analysis between relative abundance of main microbial genera and inflammatory cytokines that significantly regulated by β-carotene **(B-F)** were analyzed by Spearman’s correlation analysis. CTL, a basal diet; CARL, a basal diet containing 30 mg/kg β-carotene; CARH, a basal diet containing 90 mg/kg β-carotene. (*P < 0.05).

The correlation between the abundance of the top 40 genera and serum inflammatory cytokines concentration were presented in **Figure 5A**. Serum TNF-α concentration had a significantly positive correlation with the relative abundances of *Alloprevotella* (*P*<0.05), *Candidatus Arthromitus* (*P*<0.05), *Helicobacte*r (*P*<0.05) and *Lachnospiraceae UCG 006* (*P*<0.05), but had a negative correlation with the relative abundance of *Candidatus Stoquefichus* (*P*<0.05) and *Faecalibaculum* (*P*<0.05). Moreover, there was a significant positive correlation between serum IL-4 concentration and the relative abundance of *Helicobacter* (*P*<0.05), while serum IL-4 concentration was negatively associated with the relative abundances of *Akkermansia* (*P*<0.05), *Candidatus Stoquefichus* (*P*<0.05) and *Faecalibaculum* (*P*<0.05). The linear association of these microbial genera with serum inflammatory cytokines was shown in **Figures 5B-F**.

## Discussion

Multiple studies have focused on the effect of β-carotene on offspring, however, the impact of β-carotene on maternal and postpartum recovery is not clear. Our recent study has shown that dietary supplementation of β-carotene had a positive effect on the reproductive performance of sows (8), and *in vitro* inhibitory effect of β-carotene on cytokines was revealed by Lin et al. (25). Delivery or the release of the placenta often causes damage to the epithelium of the endometrium (26), and postpartum uterine hemorrhage is a common phenomenon (27). The present study showed that β-carotene could contribute to uterine recovery through reducing intrauterine blood stasis, with decreased uterine mass index and inflammation. Mild increase in inflammation has been considered as a normative part of healthy pregnancy, but abnormally elevated level of inflammation during pregnancy is associated with adverse birth outcomes (28). In this study, dietary supplementation of β-carotene decreased serum TNF-α and IL-4 concentration in mice after delivery. TNF-α plays a role in the mechanism of inflammation-induced preterm labor through increasing uterine smooth muscle cell collagen contractility (29, 30). TNF-α may directly promote tissue damage in pregnancy, as suggested by *in vitro* studies where TNF-α activated maternal monocytes bound to LFA-1 on placental syncytiotrophoblast and induced apoptosis (31). Immune regulation at the maternal-fetal interface is complex due to conflicting immunological objectives: protection of the fetus from maternal pathogens and prevention of immune-mediated rejection of the semi-allogeneic fetus and placenta (32). As an anti-inflammatory factor, IL-4 exerts immuno-suppressive effect by stimulating B-cells and T-cells (33). IL-4 is one of the pleiotropic anti-inflammatory cytokines that function mainly by suppressing the proinflammatory milieu (34). In this study, β-carotene decreased serum IL-4 concentration, but it has been reported that IL-4-knockout mice have normal pregnancies with respect to fetal growth and development, this would suggest that the role of an individual cytokine may not be crucial to the success of pregnancy (35). Oxidative stress can activate inflammatory cytokines (36), and our previous study demonstrated that improving antioxidant capacity can alleviate inflammation (37). Based on the relationship between inflammation and oxidation, we detected the antioxidant capacity of mice liver, but the results showed that β-carotene had no significant effect on the T-AOC and MDA content. Therefore, β-carotene exerted the anti-inflammatory effect in mice independent its antioxidant capacity. Inflammation is closely related to intestinal barrier function (38), β-carotene is mainly absorbed in the intestine, it has affinity with intestinal epithelial cells, enters the cytoplasm through the brush edge membrane, and then plays role after being catalyzed by enzymes (39). β-carotene robusts modulator of mucosal barriers (40), it improves villus height and villus height to crypt depth ratio and microbiota composition in this study, which has positive effect on the barrier function.

The microbiota play a critical role in maintaining health (41), including nutrient acquisition, immune programming, and protection from pathogens. Our previous studies have revealed that β-carotene possess an effect on gut microbiota (8), which are closely related to inflammation (22). The maternal gut microbiota undergoes dramatic changes throughout the gestation period, in which α-diversity will gradually increase throughout the lactation period (42). During pregnancy, the natural homeostasis of the gut microbiota changes toward a more inflammatory state (43), which may associate with the maternal low-grade inflammation caused by reduced fetal immune rejection. Results of the present study showed that β-carotene can significantly increase chao 1 index, observed species index and PD whole tree index, which are often used to estimate the total number of species. Specifically, β-carotene down-regulated the relative abundance of *Proteobacteria*, the microbial phylum that has been reported to be associated with increased susceptibility to colitis (44). Moreover, dietary supplementation of β-carotene improves intestinal integrity with decreased serum IL-4 concentration, which was negatively associated with the relative abundances of *Akkermansia*, a microbe genus that has been reported to increase the number of goblet cells and augment barrier integrity (45), which suggesting that the decrease of IL-4 may be related to the occurrence of low-grade inflammation in normal pregnancy. Additionally, β-carotene increased the relative abundance of genus *Candidatus Stoquefichus*, which was negatively correlated with serum TNF-α and IL-4 concentration in the present study and results by Lu et al. (46). Supplementation of β-carotene also enriched the genus *Faecalibaculum*, which contributes to butyrate production along with a shift from lactate metabolism to increased short chain fatty acids (SCFAs) production and carbohydrate metabolism (47). SCFAs mediate the transmission of signals between the microbiome and the immune system and are responsible for maintaining balance in the anti-inflammatory reaction (48). On the other hand, β-carotene reduced the relative abundance of microbial genera including *Alloprevotella* and *Helicobacter*. Correlation analysis revealed that there was a significant positive correlation between the abundance of *Alloprevotella* and serum TNF-α level, while the abundance of *Helicobacter* was positively correlated with serum levels of TNF-α and IL-4. *Alloprevotella* has been reported as producing SCFAs, and has a potential negative correlation with obesity, diabetes and metabolic syndrome (49). *Helicobacter* has a potential correlation with enteritis (50), and is considered as the majority of colonized individuals developing chronic inflammation (51). Therefore, the beneficial effects of β-carotene on inflammation may depend on a combined impact on the gut microbiota.

## Conclusions

In conclusion, dietary supplementation of β-carotene showed a positive effect on reproductive performance and postpartum recovery, with reduced postpartum hemorrhage and uterine mass index, improved villus height and villus height to crypt depth ratio, potentially by regulating inflammation-related gut microbiota. Analysis on gut microbiota revealed that β-carotene up-regulated the relative abundance of *Akkermansia*, *Candidatus Stoquefichus* and *Faecalibaculum* at the genus level, but down-regulated the relative abundance of *Proteobacteria* at the phylum level, *Helicobacteraceae* and *Peptococcaceae* at the family level*, Alloprevotella* and *Helicobacter* at the genus level. And there was a significant correlation between microbiota and serum levels of inflammatory factors.

## Data Availability Statement

The original contributions presented in the study are publicly available. This data can be found here: https://www.ncbi.nlm.nih.gov/sra/PRJNA781130.

## Ethics Statement

The animal study was reviewed and approved by Animal Care and Use Committee of Hunan Agricultural University (Permission No. 2020041).

## Author Contributions

XZY, ZH and SW are the primary investigators in this study. RH, JY, QZ, BL and XPY participated in the animal experiments. HZ and JH revised the manuscript. SW designed this study and wrote the manuscript as corresponding author. All authors contributed to the article and approved the submitted version.

## Funding

This work was partially supported by the funds from the National Natural Science Foundation of China (31741115), Fellowship of China Postdoctoral Science Foundation (2021T140715), and Hunan Provincial Natural Science Foundation for Distinguished Young Scholars (2019JJ30012).

## Conflict of Interest

Author XPY was employed by the company Hunan Xinguang’an Agricultural Husbandry Co., Ltd.

The remaining authors declare that the research was conducted in the absence of any commercial or financial relationships that could be construed as a potential conflict of interest.

## Publisher’s Note

All claims expressed in this article are solely those of the authors and do not necessarily represent those of their affiliated organizations, or those of the publisher, the editors and the reviewers. Any product that may be evaluated in this article, or claim that may be made by its manufacturer, is not guaranteed or endorsed by the publisher.
